# Expression of epithelial to mesenchymal transition-related markers in lymph node metastases as a surrogate for primary tumor metastatic potential in breast cancer

**DOI:** 10.1186/1479-5876-10-226

**Published:** 2012-11-19

**Authors:** Aleksandra Markiewicz, Tomasz Ahrends, Marzena Wełnicka-Jaśkiewicz, Barbara Seroczyńska, Jarosław Skokowski, Janusz Jaśkiewicz, Jolanta Szade, Wojciech Biernat, Anna J Żaczek

**Affiliations:** 1 Laboratory of Cell Biology, Department of Medical Biotechnology, Intercollegiate Faculty of Biotechnology, University of Gdańsk and Medical University of Gdańsk, Dębinki 1, Gdańsk, 80-211, Poland; 2 Postgraduate School of Molecular Medicine, Medical University of Warsaw, Warsaw, Poland; 3 Department of Oncology and Radiotherapy, Medical University of Gdańsk, Dębinki 7, Gdańsk, 80-211, Poland; 4 Bank of Frozen Tissues and Genetic Specimen, Medical University of Gdańsk, Dębinki 1, Gdańsk, 80-211, Poland; 5 Department of Surgical Oncology, Medical University of Gdańsk, Smoluchowskiego 17, Gdańsk, 80-214, Poland; 6 Department of Pathomorphology, Medical University of Gdańsk, Smoluchowskiego 17, Gdańsk, 80-214, Poland

**Keywords:** Breast cancer, Primary tumor, Lymph node metastasis, Gene expression, Immunohistochemistry, Biomarkers, Epithelial-mesenchymal transition

## Abstract

**Background:**

Breast cancers are phenotypically and genotypically heterogeneous tumors containing multiple cancer cell populations with various metastatic potential. Aggressive tumor cell subpopulations might more easily be captured in lymph nodes metastases (LNM) than in primary tumors (PT). We evaluated mRNA and protein levels of master EMT regulators: TWIST1, SNAIL and SLUG, protein levels of EMT-related markers: E-cadherin, vimentin, and expression of classical breast cancer receptors: HER2, ER and PgR in PT and corresponding LNM. The results were correlated with clinicopathological data and patients outcomes.

**Methods:**

Formalin-fixed paraffin-embedded samples from PT and matched LNM from 42 stage II-III breast cancer patients were examined. Expression of *TWIST1*, *SNAIL* and *SLUG* was measured by reverse-transcription quantitative PCR. Protein expression was examined by immunohistochemistry on tissue microarrays. Kaplan-Meier curves for disease-free survival (DFS) and overall survival (OS) were compared using F-Cox test. Hazard ratios (HRs) with 95% confidence intervals (95% CI) were computed using Cox regression analysis.

**Results:**

On average, mRNA expression of *TWIST1*, *SNAIL* and *SLUG* was significantly higher in LNM compared to PT (P < 0.00001 for all). Gene and protein levels of TWIST1, SNAIL and SLUG were highly discordant between PT and matched LNM. Increased mRNA expression of *TWIST1* and *SNAIL* in LNM was associated with shorter OS (P = 0.04 and P = 0.02, respectively) and DFS (P = 0.02 and P = 0.01, respectively), whereas their expression in PT had no prognostic impact. Negative-to-positive switch of SNAIL protein correlated with decreased OS and DFS (HR = 4.6; 1.1-18.7; P = 0.03 and HR = 3.8; 1.0-48.7; P = 0.05, respectively).

**Conclusions:**

LNM are enriched in cells with more aggressive phenotype, marked by elevated levels of EMT regulators. High expression of TWIST1 and SNAIL in LNM, as well as negative-to-positive conversion of SNAIL confer worse prognosis, confirming the correlation of EMT with aggressive disease behavior. Thus, molecular profiling of LNM may be used as surrogate marker for aggressiveness and metastatic potential of PT.

## Background

Distant metastasis remain the main cause of cancer-related death in breast cancer. Metastatic lymph node involvement is still the most powerful prognostic factor for relapse and death, but lymph node dissection does not affect patients survival [1]. It is still the matter of debate if positive lymph nodes are able to metastasize [2]. Currently, lymph node metastases (LNM) are considered rather a manifestation of the widespread metastatic process and a marker of aggressive phenotype of the primary tumor (PT) than the “bridge-heads” for further metastatic spread [3]. It has been confirmed by the clinical observation of poorer survival after relapse in node-positive patients compared to node-negative ones [4]. Experimental models provide further evidence that development of LNM indicates the increased potential of PT to disseminate aggressive cells and produce metastasis promoting growth factors [3], according to the recently proposed stromal progression model [5]. In this model mutual regulatory interactions between stroma and tumor cells play equally important roles in tumor progression as genetic and epigenetic changes. Those interactions contribute to the process of epithelial-mesenchymal transition (EMT), similarly to the bone marrow mesenchymal stem cells actively recruited by tumor cells to the surrounding stroma [6].

EMT has been found to be crucial in cancer dissemination, endowing cells with metastatic and cancer stem cell properties [7,8]. It is characterized by downregulation of epithelial markers (e.g. cytokeratin 8, 18, 19, E-cadherin, claudins, occludins) and upregulation of mesenchymal markers (e.g. vimentin, N-cadherin) [9], what results in numerous phenotypic changes such as the loss of cell-cell adhesion and cell polarity, and the acquisition of migratory and invasive properties [10,11]. TWIST1, SNAIL and SLUG are transcription factors among those governing EMT (EMT-TFs) [10]. Increased expression of EMT-TFs examined in PT has been associated with poor prognostic clinico-pathological features and outcome in breast and other cancers [12-14] as well as multidrug resistance [15]. However, to the best of our knowledge there are no data available on the status of TWIST1, SNAIL and SLUG in LNM.

Phenotype of the aggressive cancer cells subpopulations resulting from EMT might not be easily captured in the PT since they form a minority of cells within tumor bulk [16-18]. Moreover, expression of genes associated with EMT is transient and space-limited [19]. We hypothesized that the analysis of pre-selected subpopulations of cancer cells found in LNM could be more informative in terms of aggressiveness than the analysis of the PT bulk. The primary objective of the present study was to evaluate possible changes occurring in classical and EMT-related marker status between PT and corresponding synchronous axillary LNM before initiation of the therapy and to relate them to clinical outcome. Additionally, we aimed to investigate the feasibility of the quantitative PCR-based gene expression profiling of low-level transcripts *TWIST1*, *SNAIL* and *SLUG* in formalin-fixed, paraffin-embedded (FFPE) tissues compared to matched frozen counterparts.

## Material and methods

### Tissue specimens

Studied material included 44 tissue specimens from patients with operable breast cancer and lymph node involvement who were treated between 2006–2008 at the Medical University of Gdansk Hospital. Patients were treated with surgery by modified radical mastectomy or local tumor resection, with axillary node dissection followed by postoperative breast irradiation. Adjuvant therapy with chemotherapy and/or hormone therapy was given in standard care settings based on the nodal and hormone receptor status. Availability of PT and matched LNM was mandatory. Patients with no evidence of lymph node involvement or earlier chemotherapy were not eligible for this study. Non-cancer control breast tissue samples were acquired during mastectomy ensuring the greatest possible distance to the main tumor mass, and sections of non-involved lymph nodes were collected. The study was conducted in accordance with the Declaration of Helsinki and approved by the Ethics Committee of the Medical University of Gdansk. All patients signed informed consent forms.

### RNA extraction from formalin-fixed paraffin-embedded (FFPE) tissue

Tissue specimens were fixed in 10% (v/v) neutral-buffered formalin for up to 24 h, dehydrated in 70% ethanol and embedded in paraffin. FFPE tissue blocks were stored at room temperature for up to 6 years. The percentage of tumor cells in each FFPE specimen was evaluated by hematoxylin and eosin staining reviewed by a certified pathologist. Only the tissue section with confirmed presence of invasive carcinoma and tumor cells content over 50% were included. 2–4 slices of 10 μm thickness were cut using a microtome and placed in 1.5 ml centrifuge tubes. Tissues were de-paraffinized by treatment with xylene and 100% ethanol. Total RNA was isolated using RNeasy FFPE Kit (Qiagen, Germany) according to the manufacturer's protocol, including on-column DNase I treatment.

### RNA extraction from fresh-frozen (FF) tissue

After collection, tissue samples were immediately frozen in liquid nitrogen and stored at −80°C for further analysis. 20–30 mg tissue sections were homogenized with zircon beads in MagNA Lyzer (Roche, Germany) for 40 s. Total RNA was isolated using RNeasy Mini Kit (Qiagen, Germany) according to the manufacturer’s protocol, including on-column DNase I treatment.

### RNA analysis and reverse transcription

For all samples RNA concentration and purity was determined using the Nano-Drop ND-1000 spectrophotometer (Thermo Scientific, USA). Qualitative analysis of RNA was performed by microcapillary electrophoresis using the Agilent 2100 Bioanalyzer (Expert software version B.02.08) with an RNA Nano Chip (Agilent Technologies, UK). For each sample, whenever possible, 1 μg of RNA was used as the template in the reverse transcription reaction using Transcriptor First Strand cDNA Synthesis Kit (Roche, Germany) in a 20 μl volume with random hexamer primers, according to the manufacturer's protocol.

### qPCR and gene expression analysis

Gene expression levels were determined by RT-qPCR on the CFX96 Real-Time System (Bio-Rad, USA) with specific TaqMan Gene Expression Assays (Applied Biosystems/Life Technologies, USA) containing validated primers and probe set spanning exon-exon boundaries for detection of *TWIST1* (Hs00361186_m1), *SNAIL* (Hs00195591_m1) and *SLUG* (Hs00950344_m1). Relative expression values of each gene were calculated by the delta-delta-C_t_ method normalized to the reference gene *GAPDH* (glyceraldehyde 3-phosphate dehydrogenase, Hs99999905_m1), and non-cancer breast tissue as a calibrator with the use of qBasePLUS software (Biogazelle, ver. 2.0).

Stability of four reference genes: *β-actin* (Hs99999903_m1), *HPRT* (Hs99999909_m1), *GAPDH* and *YWHAZ* (Hs03044281_g1) was evaluated in 11 breast cancer samples. *GAPDH* was chosen as a reference gene based on its highest expression stability measure (M) calculated in GeNorm.

The qPCR cycling profile was programmed as follows: predenaturation at 95°C for 10 minutes, followed by amplification phase, which included denaturation at 95°C for 15 seconds, annealing and extension at 60°C for 60 seconds for 45 cycles. 40 ng of cDNA in 4 μl was added per reaction and mixed with 10 μl of TaqMan Universal PCR Master Mix (Applied Biosystems/Life Technologies, USA), 5 μl of water and 1 μl of specific primer and probe mix (20x concentrated). To verify that the qPCR signals derived from cDNA, not genomic DNA, for each gene tested a control without reverse transcriptase in the RT reaction (no RT control) was included. Each sample was analyzed in duplicate, and the average C_t_ value of duplicates was used as a quantitative value. The specificity of the polymerase chain reaction was confirmed by gel electrophoresis using 1.5% agarose gel containing Gel-Red (Gentaur, Belgium) and viewed under ultraviolet illuminator Gel Doc (Bio-Rad, USA). Positive result was defined when a relative gene expression was higher than median expression in all tumor samples.

### RT-qPCR standardization/validation

To evaluate the suitability of the low-level transcripts expression analysis in FFPE samples, we standardized the method and compared it with the gold-standard – the expression in frozen samples. To ensure RT-qPCR experiments’ relevance and accuracy, analytical sensitivity was measured in accordance with MIQE (Minimum Information for Publication of Quantitative Real-Time PCR Experiments) guidelines [20]. For 30 matched frozen and FFPE primary tumors the following parameters were evaluated for the *TWIST1* gene, as it has been expressed at lowest levels throughout all our experiments:

1. Efficiency of the reaction

2. Sensitivity: limit of detection (LOD) and limit of quantification (LOQ)

3. Intra- and interassay variation

Detailed description of the standardization methodology is described in the Additional file 1.

### Immunohistochemistry (IHC) on tissue microarrays (TMA)

TMAs were prepared as described before [21]. Protein expression was examined using antibodies against TWIST1 [ab50581, Abcam], SNAIL [ab85936, Abcam], SLUG [ab38551, Abcam], E-cadherin [Nch 38, Dako], vimentin [V9, Dako], HER2 [HercepTest Kit, Dako], ER [1D5, Dako], PgR [636, Dako] and peroxidase-based detection system (Novolink Polymer Detection System, Novocastra, UK) in accordance with the manufacturer’s guidelines. Antigen retrieval was carried out by heat induced epitope retrieval at pH 6. Positive and negative controls for each marker were used according to the supplier’s data sheet. The material was analyzed using a transmission light microscope (Olympus BX 41) with 400x magnification. Two cores from each tumor were assessed individually. IHC analysis was performed by two independent observers blinded to the clinical data and patients outcomes. Discordant results were reviewed to achieve agreement. The same protocol of staining and scoring was used for both PT and LNM. There was half a year difference in staining of PT and LNM.

ER and PgR were scored according to classical Allred system with cut-point 3 for positive result, while HER2 - according to HercepTest criteria, with 3+ score defining positive result. For vimentin and E-cadherin positive result was considered when 10% or more cells stained positively [22,23]. For TWIST1, SNAIL and SLUG only nuclear staining was considered with a 10% cut off value of positivity [22-24]. Results were considered concordant if PT and LNM were both positive or both negative, other combinations were considered discordant and denoted the conversion rate.

### Statistical analysis

To fulfill the primary objective of determining the probability of conversion in biomarker status, 28 paired samples were required to detect a discordance rate of 20% with 80% power using a one-sided alpha of 5%.

Concordance between PT and LNM was measured by estimating Cohen’s kappa coefficient (κ) with Medcalc software, version 12.2.1.0 (MedCalc Software, Belgium). The level of agreement based on κ values was assessed using the Landis and Koch criteria: 0.00-0.20, slight agreement; 0.21-0.40, fair agreement; 0.41-0.60, moderate agreement; 0.61-0.80, substantial agreement; and 0.81-1.00, almost perfect agreement [25].

Categorical variables were compared by Fisher’s exact test, and continuous variables were compared by the Spearman’s rank order test. Kaplan-Meier curves for disease-free survival (DFS) and overall survival (OS) were compared using F-Cox test. DFS was defined as the time from surgery to an event (local or distant relapse, second malignancy or death, whichever came first) or censoring. A censoring was defined as lost to follow-up or alive without relapse at the end of follow-up. Hazard ratios (HRs) with 95% confidence intervals (95% CI) were computed using Cox regression analysis. Significance was defined as P ≤ 0.05. STATISTICA software version 10.0 for Windows was used for all statistical analyses.

## Results

The median age of the patients was 56.5 years (Table 1). The estimated median follow up, as calculated by the reverse Kaplan-Meier method [26], was 4.2 years. The median follow up of patients who did not have an event (n = 34) was 4.1 years, and those with an event – 2.6 years (n = 10). The average number of metastatic lymph nodes was 5.2 (range 1–27).

**Table 1:** Patients characteristics

**Variable**	**Number of cases N = 44**	**%**
**Age (years)**		
median	56.5	
range	33-77	
**T stage**		
T1-2	38	86.4
T3-4	6	13.6
Missing data	-	
**N stage**		
N1	21	48
N2	18	41
N3	5	11
Missing data	-	
**ER status**		
Negative	17	38.6
Positive	25	56.8
Missing data	2	4.6
**PgR status**		
Negative	15	34.1
Positive	27	61.3
Missing data	2	4.6
**HER2 status**		
Negative	24	54.5
Positive	12	27.3
Missing data	8	18.2
**Histological type**		
Ductal	34	77.3
Lobular	6	13.6
Other	4	9.1
Missing data	-	
**Grade**		
G1-2	36	81.8
G3	8	18.2
Missing data	-	

**Figure 1:**
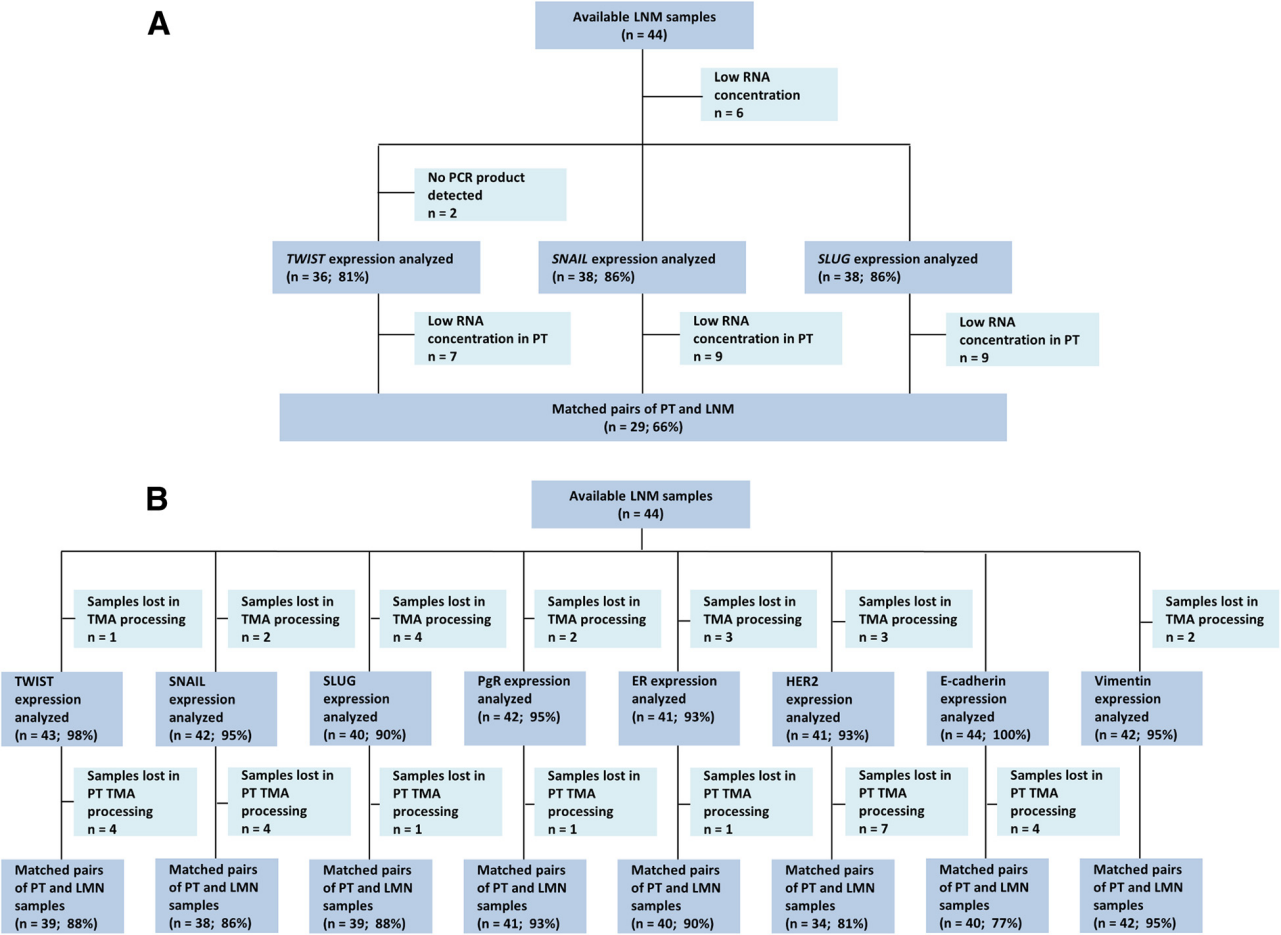
**Flow chart of samples analyzed with A) RT-qPCR and B) IHC.** Abbreviations: PT – primary tumor, LNM – lymph node metastasis, TMA – tissue microarrays, ER – estrogen receptor, PgR – progesterone receptor, n – number of cases.

Of the 44 paired PT and LNM, 29 pairs (66%) had amplifiable RNA (Figure 1A). Relative expression of *TWIST1*, *SNAIL* and *SLUG* was significantly higher in LNM compared to PT: 1.75 ± 2.41 vs. 0.25 ± 0.32 for *TWIST1* (P < 0.00001), 2.8 ± 3.06 vs. 0.96 ± 2.32 for *SNAIL* (P < 0.00001), 1.03 ± 0.93 vs. 0.16 ± 0.19 for *SLUG* (P < 0.00001). The conversion rates in *TWIST1*, *SNAIL* and *SLUG* mRNA status between PT and paired LNM were 52%, 28% and 45%, respectively (Table 2). Detailed representation of all the analyzed markers conversion rates is shown in Additional file 2: Table S1.

**Table 2:** Biomarkers’ conversion rate between paired PT and LNM

**Marker**	**N**	**Positive in PT**	**Positive in LNM**	**Conversion rate PT → LNM**			
		**N (%)**	**N (%)**	**(−) → (+) N (%)**	**(+) → (−) N (%)**	**N (%)**	**kappa coefficient (95% CI)**
**mRNA *TWIST1***	29	14 (48)	17 (59)	9 (31)	6 (21)	15 (52)	−0.03 (−0.38-0.33)
**mRNA *SNAIL***	29	16 (55)	14 (48)	3 (10)	5 (17)	8 (28)	0.45 (0.13-0.77)
**mRNA *SLUG***	29	14 (48)	15 (52)	6 (21)	7 (24)	13 (45)	0.1 (−0.26-0.47)
**TWIST1**	39	15 (38)	17 (44)	10 (26)	8 (20)	18 (46)	0.05 (−0.26-0.36)
**SNAIL**	38	15 (39)	29 (76)	17 (45)	3 (8)	20 (53)	0.05 (−0.18-0.29)
**SLUG**	39	10 (26)	7 (18)	7 (18)	4 (10)	11 (28)	0.18 (−0.16-0.52)
**Vimentin**	42	5 (12)	4 (10)	1 (2)	2 (5)	3 (7)	0.63 (0.24-1)
**E-cadherin**	40	35 (87)	35 (87)	0 (0)	0 (0)	0 (0)	1 (1.0-1.0)
**ER**	40	23 (57)	28 (70)	6 (15)	1 (3)	7 (18)	0.63 (0.39-0.87)
**PgR**	41	26 (63)	32 (78)	9 (22)	3 (7)	12 (29)	0.31 (0.02-0.6)
**HER2**	34	5 (15)	6 (18)	1 (3)	0 (0)	1 (3)	0.89 (0.68-1)

Conversion described as the number (percentage) of discordant cases and kappa coefficient of concordance.

IHC staining was successful for paired samples of PT and LNM for TWIST1, SNAIL and SLUG in 88%, 86% and 88% of cases (Figure 1B). Cores were missing, folded, or contained no invasive cancer in 12%, 14%, and 12%, respectively. Exemplary photographs of immunohistochemical staining of lymph nodes metastases are presented in Additional file 3: Figure S3 and in Additional file 4: Figure S4.

The conversion rates in TWIST1, SNAIL and SLUG protein status between PT and paired LNM were 46%, 53% and 28%, respectively (Table 2). Negative-to-positive conversion of all TF occurred more frequently than positive-to-negative one (Table 2).

IHC staining of TWIST1 protein was concordant with expression levels of *TWIST1* mRNA measured by RT-qPCR in 83% (P = 0.002). No correlation was observed for other TFs. The conversion rates of E-cadherin and vimentin were much lower (0% and 7%, respectively), while the HER2, ER and PgR conversion occurred in 3%, 18% and 29% of cases, respectively.

Expression of TWIST1, SNAIL and SLUG on mRNA and protein level in PT and LNM was correlated with the number of involved lymph nodes. Increased number of involved lymph nodes (more than 3) correlated with elevated expression of TWIST1 protein in LNM (P = 0.02) and showed a trend towards increased expression of *TWIST1* mRNA in LNM (P = 0.07) and SNAIL protein in LNM (P = 0.07) (in Additional file 5: Table S2).

**Figure 2:**
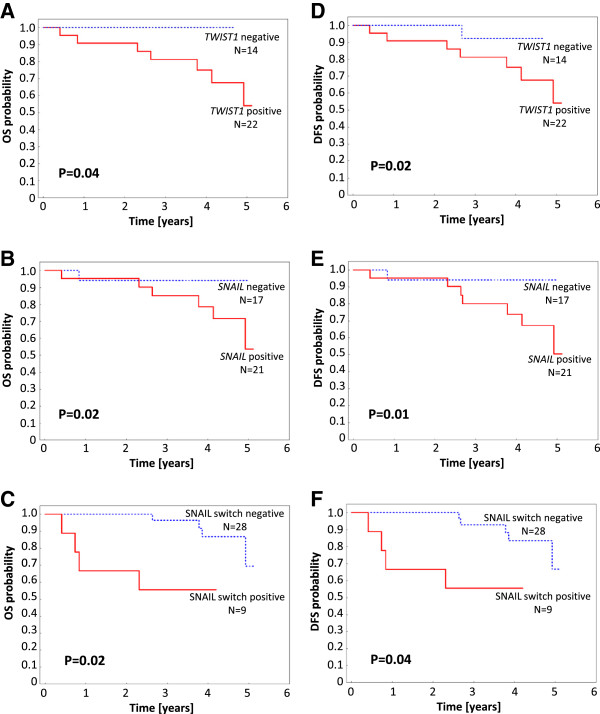
**Kaplan-Meier curves according to biomarker status.** Overall survival (A, B, C) and disease-free survival (D, E, F) for different *SNAIL* (B, E) or *TWIST1* (A, D) mRNA status in LNM and SNAIL (C, F) protein switch status from negative in PT to positive in LNM.

Increased mRNA expression of *TWIST1* and *SNAIL* in LNM was associated with shorter OS (P = 0.04 and P = 0.02, respectively) and DFS (P = 0.02 and P = 0.01, respectively), whereas their expression in PT had no prognostic impact (Figure 2). Negative-to-positive switch of SNAIL protein correlated with shorter OS and DFS (P = 0.02 and P = 0.04, respectively) (Figure 2). SNAIL protein negative-to-positive switch was associated with significantly increased HR for both OS (HR = 4.6; 1.1-18.7; P = 0.03) and DFS (HR = 3.8; 1.0-48.7; P = 0.05). Conversion of ER, PgR and other biomarkers had no significant impact on survival.

### RT-qPCR standardization results

**Figure 3:**
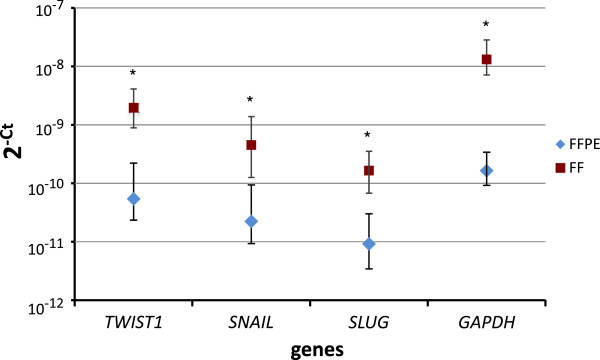
**Expression level of *TWIST1*, *SNAIL*, *SLUG *and *GAPDH *in FFPE and FF specimen presented as two to the power of minus Ct (2^-Ct^).** Asterisk (*) indicates statistically significant difference (p < 0.0001) in average expression levels between FF and FFPE samples. Bars represent standard errors. As recommended by Livak and Schmittgen [57] statistical analysis and errors were calculated on transformed (2^-Ct^) data instead raw Ct values.

RNA extracted from FF material had better quality than from FFPE, as presented by higher mean RIN 9.5 ± 0.6 vs. 2.1 ± 0.5 (Table 3 and in Additional file 1: Figure S1). The efficiencies of the post-PCR *TWIST1* standard amplification reactions were comparable: 95.5% for FF and 94.3% for FFPE material. For LOD and LOQ calculation the following values were taken: slopes of the generated standard curves (in Additional file 1: Figure S2) for relative *TWIST1* expression level in FF (0.977) and FFPE (1.218) and SD of *TWIST1* relative expression level in FF (0.014) and FFPE (0.018). In case of FFPE tissues *TWIST1* LOD was 0.048 and LOQ 0.147, for FF tissues 0.043 and 0.143, respectively. Intra- and interassay variation in FF samples was: 9.83% and 12.96%, respectively. For FFPE samples intra- and interassay variation equaled 21.65% and 23.34%, respectively. The expression level of each gene was significantly higher in the FF tissues than in FFPE tissues (Figure 3). For *SNAIL* and *SLUG* the expression correlated between FFPE and corresponding FF samples (R = 0.58, P = 0.001; R = 0.44, P = 0.02, respectively). For *TWIST1* the correlation was borderline (R = 0.35, P = 0.07).

**Table 3:** Comparison of RNA quantity, quality and RT-qPCR performance for FF and FFPE derived templates

	**FF**	**FFPE**	**P value**
RNA yield [ng/1 mg tissue]*	210 ± 217	227 ± 159	NS
**median 260/280 ratio**	1.97	1.98	
RIN*	9.5 ± 0.6	2.1 ± 0.5	<0.0001
**LOD**	0.043	0.048	
**LOQ**	0.143	0.147	
**efficiency of qPCR**	95.5%	94.3%	
average Ct*	29.9 ± 2.1	35.4 ± 2.7	<0.0001
**interassay variation CV**	12.96%	23.34%	
**intraassay variation CV**	9.83%	21.65%	

* Data are given as mean ± standard deviation.

Abbreviations: *FF* fresh frozen specimens, *FFPE* formalin-fixed paraffin-embedded specimens, *RIN* RNA integrity number, *LOD* limit of detection, *LOQ* limit of quantification, *CV* coefficient of variation, *NS* not significant.

## Discussion

Despite the years of routine use of lymph node dissection in breast cancer management, inspired by Halstedt [27], our understanding of the role of lymph nodes in the metastatic process is still marginal. The analysis of pre-selected subpopulations of cancer cells found in lymph nodes metastases could provide insights into biological background of cancer progression. Our work explored patterns of conversion in classical and EMT-related biomarker status between primary breast tumors and corresponding synchronous axillary lymph node metastases to determine whether phenotypic variability is associated with different clinical outcome.

Numerous studies have shown discordant expression of classical molecular markers, ER, PgR and HER2 between the PT and both lymph nodes and distant metastases [28-32]. Discordance in ER and PgR status has been demonstrated within the range of 10-32% and 34%–41% cases, respectively [28,33-35] and 3%–24% for HER2 status [28,32,34-36] when PT were compared with metastatic relapse. Generally, lower discordance rates have been observed in LNM, as showed in individual studies [30,31,37] and confirmed in recent meta-analysis on HER2 status in primary and metastatic cancer, including 26 studies of 2520 subjects [38]. However, there are also reports showing the opposite, as exemplified by the study of Aitken *et al.*, who found different breast/node status of at least one receptor (ER, PgR or HER2) in almost 47% of cases [29].

Receptor discordance has been frequently associated with poor survival [32,34,35], what has been attributed to the inappropriate use of hormone and targeted therapy prescribed based on the characteristics of PT [35] or selection of tumors with more unstable phenotype and therefore more aggressive behavior [28]. Adverse impact of receptor discordance was abolished, when treatment was modified according to the results of metastatic biopsy [28]. In general, the cancer management scheme in 14-20% patients was changed on the basis of metastasis biopsy [28,33,39]. The growing body of evidence supports reassessment of ER, PgR and HER2 at the time of relapse diagnosis to tailor the most effective treatment for each patient at all times [40].

Until now, main research focus has been put on the discordance of ER, PgR and HER2 status between the PT and metastasis, as discussed above. Some studies examined cell proliferation, differentiation and apoptosis markers [30,37,41]. However, to the best of our knowledge there is no data available on the status of EMT regulators: TWIST1, SNAIL and SLUG in LNM compared to PT. The clinical outcome in relation to these biomarker status in lymph nodes has not yet been reported.

Our results show frequent conversion of TWIST1, SNAIL and SLUG status between PT and LNM, occurring both at mRNA and protein level (within the range of 28-53% of cases). Expression levels of EMT-TFs were significantly higher in LNM compared to PT. This could be explained by the higher frequency of pre-selected aggressive subpopulations of cancer cells resulting from EMT present in LNM than in the PT, where these aggressive cells constitute a minority of cells [16,17] and therefore might not be easily captured. It seems that indeed molecular profile of LNM might be a surrogate marker of aggressive phenotype of the PT, as postulated [3].

We have found that both the elevated level of TFs in LNM and their negative-to-positive switch were associated with poor clinical outcome. Increased *TWIST1* and *SNAIL* expression in LNM correlated with shorter OS (P = 0.04 and P = 0.02, respectively) and DFS (P = 0.02 and P = 0.01, respectively), whereas their expression in PT had no prognostic impact. Negative-to-positive conversion of SNAIL status also correlated with worse survival compared to unchanged status (OS: HR = 4.6; 1.1-18.7; P = 0.03; DFS: HR = 3.8; 1.0-48.7; P = 0.05). Numerous studies show poor prognostic impact of increased expression of TWIST1, SNAIL and SLUG in PT [12-14,42-44]. The contradictory results have also been reported [24]. But no study have examined their clinical outcome in lymph nodes until now.

We also described the protein status of EMT markers – E-cadherin and vimentin in PT and LNM. We have observed no or little change in E-cadherin and vimentin status between PT and LNM. Our results and the results of other groups [45] show that E-cadherin is at least as frequently expressed in LNM as in primary tumors and it is not downregulated in LNM, what would be expected if EMT was involved. However, this observation does not exclude the possibility that E-cadherin undergoes transient downregulation during EMT and is re-expressed at the stage of circulating tumor cells to facilitate adhesion and metastases formation [46]. Similar to other groups we observed that vimentin is rarely expressed in LNM [47] or is reduced in comparison to the PT [48]. However, expression of vimentin in metastases was also shown to be heterogeneous, metastasis size and side dependent [49], what supports the notion that vimentin expression is transient and environment-controlled. As the observed change in EMT-TFs status between PT and LNM is a new finding, a question arises if it results from true biological variation or from inconsistent measurement.

There are many hypotheses that could explain biomarkers change between PT and corresponding metastasis at biological level. Clonal selection, with subsequent clonal expansion during tumor progression, has been proposed as the mechanism inducing the differences in the genetic composition of primary and metastatic breast cancer [50,51]. Clonal selection may be related to intra-tumor heterogeneity [52,53] and/or to various selective pressures such as the immune surveillance of the host, stromal or growth factor interactions, nutritional deficiencies, hypoxia, and therapy. Indeed, Niikura reported recently a significantly higher discordance rate in HER2 status among women who received chemotherapy than among those who did not [32]. To the biological variation between primary and metastatic tumor could also contribute independent evolution of an early stem cell clones in both sites, instead of a linear progression from the PT to metastasis [54].

The discordance between PT and LNM may also be caused by inconsistent measurement resulting from numerous technical issues [55]. For example, recent meta-analysis revealed 15 pre-analytical variables capable of impacting IHC, including fixation delay, fixative type, time in fixative, reagents and conditions of dehydration, clearing, and paraffin impregnation, and conditions of slide drying and storage [56]. Analytical procedures of antigen retrieval, immunostaining and interpretation of results add additional variance to the final result [55]. To ensure maximal consistency, in our study all specimens were from single cancer hospital, examined by the same two observers with the same protocol of staining and scoring for PT and LNM. Additionally, independent observers were blinded to the clinical data and patient outcome.

The results should be, however, interpreted with caution because there is a possibility of selection bias due to retrospective character of the study. Another limitation might be the small sample size. But even with that number of patients the study had still sufficient power to detect reliably the 20% difference in biomarker status, and all of the discussed differences are above that value. It has been claimed that to reduce reporting errors the use of confirmatory test is recommended [40]. To strengthen our study we confirmed increase of EMT-TFs at both mRNA and protein level with RT-qPCR and IHC, respectively. Taking into consideration discussed biological and technical issues, it seems that current study demonstrates true biological variation in TWIST1, SNAIL and SLUG in PT and LNM.

To make gene expression most reliable we have carefully validated the method we used. Since gene expression profiling in lymph nodes could only be examined in formalin-fixed, paraffin-embedded (FFPE) material, we have undertaken the methodological substudy to assess the reliability of obtained results of RT-qPCR. We aimed to investigate the feasibility of RT-qPCR-based gene expression profiling of low-level transcripts *TWIST1*, *SNAIL* and *SLUG* in FFPE tissues compared to matched frozen counterparts.

Expression of genes associated with EMT is transient and space-limited [19], what makes them specially difficult to study in clinical setting. Technical difficulties that interfere with reliable gene expression analysis can be particularly prominent in FFPE samples, in which RNA is degraded and chemically modified, resulting in lower RNA Integrity Number (RIN) values and rendering it inaccessible for amplification.

In order to examine to what extent tissue fixation influences RT-qPCR based methods we have measured sensitivity of the method in use by defining limits of detection and quantification (LOD and LOQ). No sample presented relative gene expression value of *TWIST1* lower than calculated LOD and LOQ, which confirms the reliability of our data, as experimental results less than the theoretically possible LOD should never be reported.

The overall profile of *TWIST1, SNAIL* and *SLUG* expression was similar to that generated with well-preserved RNA from matched FF tissue. We presume however, that decreased functionality of RNA (due to degradation or modification) is the underlying factor responsible for increased intra- and interassay variation performed on FFPE samples. This process did not affect the sensitivity of the method as the assay measures relative quantity of the gene expression, which does not change due to similar degree of reference gene and examined genes degradation.

Thus, despite low RIN values RNA from FFPE samples may work fine in quantitative PCR in terms of specificity, sensitivity and reproducibility, even for low-level transcripts, as exemplified by *TWIST1*, *SNAIL* and *SLUG*.

## Conclusions

In conclusion, this is the first study to date demonstrating that samples derived from LNM are enriched in cells with more aggressive phenotype marked by elevated levels of EMT regulators. High expression of these markers correlates with increased number of involved lymph nodes and decreased survival of breast cancer patients. We postulate that examination of molecular profile of lymph node metastasis could provide information about aggressiveness potential of the PT and the characteristics of seeded cells. It therefore has the potential to be developed into a powerful prognostic biomarker in breast cancer, ensuring an accurate prognosis and treatment selection. A prospective study of larger cohort of patients would be necessary to confirm the clinical significance of the changes in EMT-related molecular markers.

## Abbreviations

DFS: Disease-free survival; EMT: Epithelial-to-mesenchymal transition; ER: Estrogen receptor; FF: Fresh-frozen specimen; FFPE: Formalin-fixed, paraffin-embedded specimen; HER2: Human epidermal growth factor receptor 2, LNM, lymph nodes metastases; OS: Overall survival; PgR: Progesterone receptor; PT: Primary tumor; RIN: RNA integrity number; TMA: Tissue microarray; TF: Transcription factor.

## Competing interests

The authors declare that they have no competing interests.

## Authors’ contributions

AM designed methods and experiments, standardized/validated RT-qPCR, participated in the data interpretation, drafted the manuscript. TA carried out gene expression analysis, standardized/validated RT-qPCR, performed statistical analysis, participated in the data interpretation, helped to draft the manuscript. MWJ participated in the assembly of the clinical data and the data interpretation, performed statistical analysis. BS provided samples from breast cancer patients, participated in the assembly of the clinical data. JS designed and performed immunohistochemical analyses of tissue microarrays. JJ provided samples from breast cancer patients, participated in the assembly of the clinical data. JS provided samples from breast cancer patients, participated in the assembly of the clinical data. WB performed immunohistochemical analyses of tissue microarrays, participated in the data interpretation. AJZ defined the research theme, designed methods and experiments, participated in the data interpretation, performed statistical analysis, drafted the manuscript. All authors read and approved the final manuscript.

## Supplementary Material

Additional file 1 **Figure S1.** Qualitative analysis of RNA from matched FFPE and FF samples. In microcapillary electrophoresis FF tissues afforded a clear band of complete fragments derived from 28S and 18S rRNA. The RNA fragments in FFPE are highly degraded. Samples from five representative tumors are shown. Figure S2. Standard curves for *TWIST1* relative expression levels in: A) FF and B) FFPE tissues. Click here for file

Additional file 2 **Table S1.** Summary of the examined marker conversion status in matched LNM and PT. Green, cases with negative-to-positive switch in LNM versus PT; red, positive-to-negative switch in LNM compared to PT, yellow no change in expression; white, unable to analyze sample. Click here for file

Additional file 3 **Figure S3.** Immunohistochemical staining of EMT-related markers in lymph nodes metastases. Exemplary results of negative and positive staining of E-cadherin, vimentin, TWIST1, SLUG and SNAIL. Click here for file

Additional file 4 **Figure S4.** Immunohistochemical staining of receptors in lymph nodes metastases. Exemplary results of negative and positive staining of estrogene receptor (ER), progesterone receptor (PgR) and human epidermal growth factor receptor 2 (HER2). Click here for file

Additional file 5 **Table S2.** Correlation between the expression of EMT-transcription factors and the number of involved lymph nodes. Results for both mRNA and protein levels of TWIST1, SNAIL and SLUG are presented. Click here for file
