# Supplementary material for: Expression of epithelial to mesenchymal transition-related markers in lymph node metastases as a surrogate for primary tumor metastatic potential in breast cancer
Source: J Transl Med. 2012 Nov 19;10:226. doi: 10.1186/1479-5876-10-226 (PMC3524044; doi:10.1186/1479-5876-10-226)
Supplement: Additional file 1 — Figure S1. Qualitative analysis of RNA from matched FFPE and FF samples. In microcapillary electrophoresis FF tissues afforded a clear band of complete fragments derived from 28S and 18S rRNA. The RNA fragments in FFPE are highly degraded. Samples from five representative tumors are shown. Figure S2. Standard curves for TWIST1 relative expression levels in: A) FF and B) FFPE tissues. [file 1479-5876-10-226-S1.pdf]

RT-qPCR validation/standardization – methodology and results

Methodology

1. Efficiency of the qPCR reaction

To prepare a standard, *TWIST1* transcript was amplified in a PCR reaction using external primers designed to amplify a region of 392 bp encompassing fragment detected in qPCR reaction with TaqMan Gene Expression Assay. The sequences of the primers were F-5'-GACTTCCTCTACCAGGTCCTCCAG-3' and R-5'-AGGATGGTGCCGCTGCCCGTCTG-3'. Primers were designed with Molecular Beacon software (Premier Biosoft ver. 7.9). Amplification was performed in Mastercycler Gradient (Eppendorf, Hamburg, Germany) in 25 µl volume using 200 nM of each primer (Sigma), 2 mM MgCl2, 200 µM of each dNTP, 1U of GoTaq Flexi Polymerase (Promega, Madison, USA), and 100 ng of cDNA in a volume of 4 µl. PCR steps were set to 2 min at 95 °C, followed by 35 cycles of denaturation at 94 °C for 20 s, annealing at 62 °C for 30 s, extension at 72 °C for 40 s, and final extension 72 °C for 7 min. Specificity of the product was checked on 2.5% agarose gel stained with ethidium bromide.

2. Sensitivity

In order to determine limit of detection (LOD) and limit of quantification (LOQ) relative expression level of *TWIST1* gene was measured in the standards prepared by six 10-fold serial dilutions of the post-PCR *TWIST1* product spiked into 20 ng of cDNA prepared from breast tissue sample without *TWIST1* expression. Plotting *TWIST1* relative gene expression levels against serial dilution factor of the post-PCR *TWIST1* solution, allowed for calculation of the slope for LOD and LOQ determination according to the equations:

a. LOD=3.3*SD/slope

b. LOQ=10*SD/slope

SD values were calculated from 10 duplicate measurements of *TWIST1* relative expression level in the smallest standard giving stable Ct values for *TWIST1* (difference below 0.5 cycle). Analyses were performed separately for FF and FFPE tissues (cDNA from either FF or FFPE was used in the experiments), using the same post-PCR TWIST1 standard.

3. Intra- and interassay variation

Intraassay variation was measured as the coefficient of variation (CV) in the relative *TWIST1* expressions level in a breast tissue. Five samples (technical replicates) were analyzed in duplicates in one experiment. This experiment was repeated by another analyst at a different time point in order to calculate the CV for interassay variation evaluation. The analysis was performed separately for FF and FFPE samples.

Results

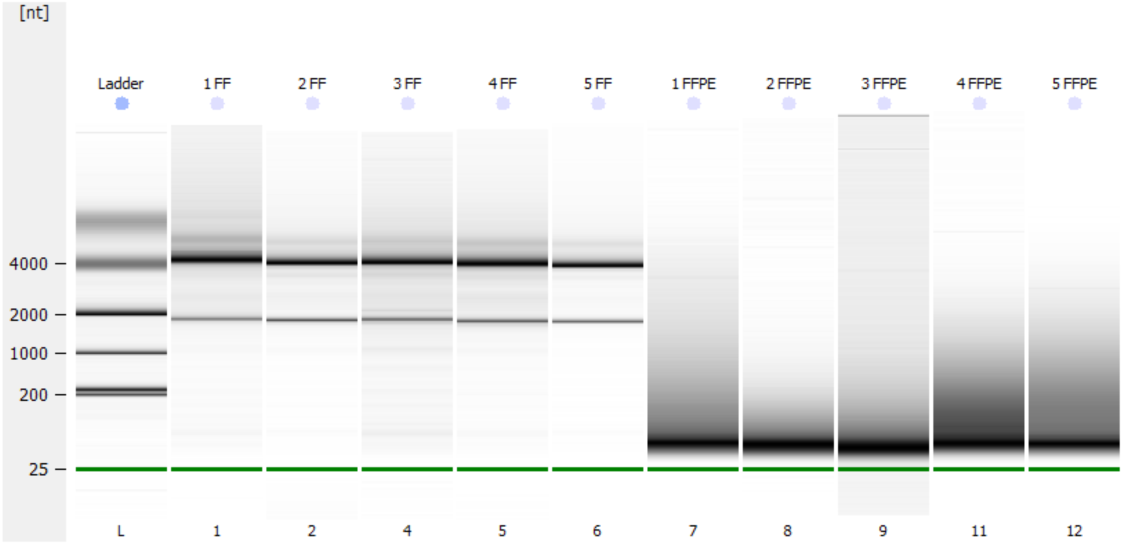

Figure 1S. Qualitative analysis of RNA from matched FFPE and FF samples. In microcapillary electrophoresis FF tissues afforded a clear band of complete fragments derived from 28S and 18S rRNA. The RNA fragments in FFPE are highly degraded. Samples from five representative tumors are shown.

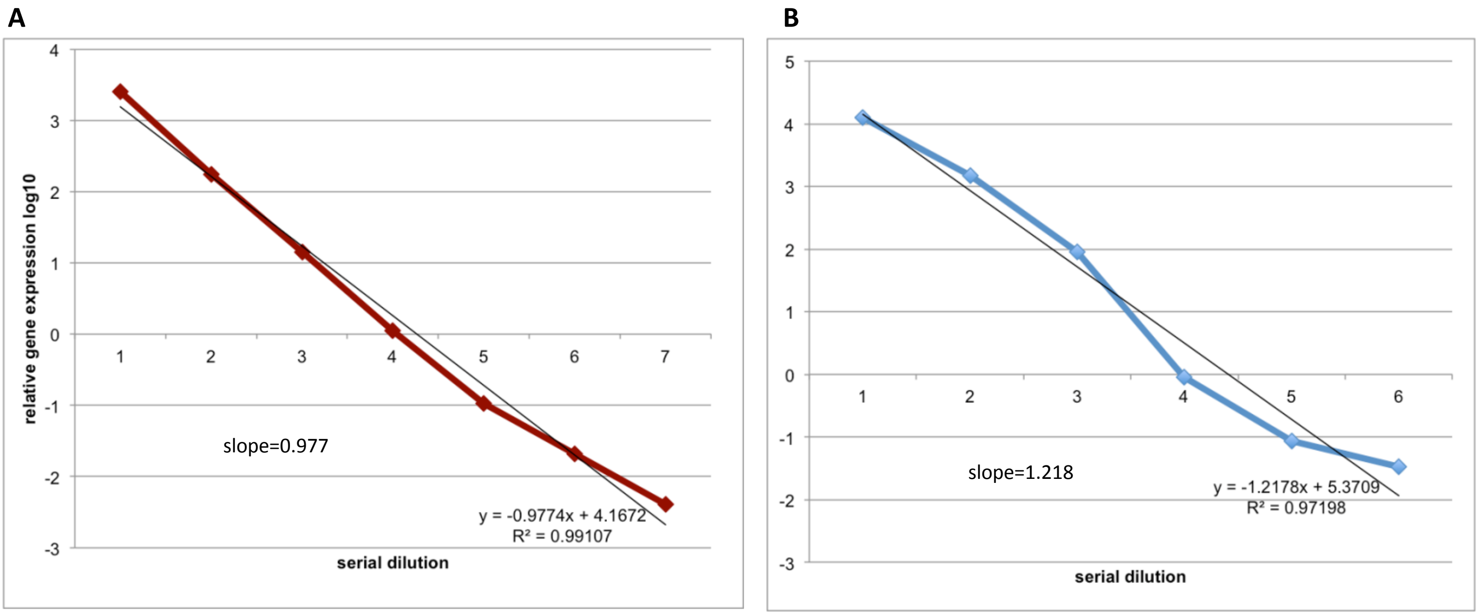

Figure 2S. Standard curves for *TWIST1* relative expression levels in: A) FF and B) FFPE tissues.
