# Supplementary material for: Expression of epithelial to mesenchymal transition-related markers in lymph node metastases as a surrogate for primary tumor metastatic potential in breast cancer
Source: J Transl Med. 2012 Nov 19;10:226. doi: 10.1186/1479-5876-10-226 (PMC3524044; doi:10.1186/1479-5876-10-226)
Supplement: Additional file 2 — Table S1. Summary of the examined marker conversion status in matched LNM and PT. Green, cases with negative-to-positive switch in LNM versus PT; red, positive-to-negative switch in LNM compared to PT, yellow no change in expression; white, unable to analyze sample. [file 1479-5876-10-226-S2.pdf]

Table 1S. Summary of the examined marker conversion status in matched LNM and PT. Green, cases with negative-to-positive switch in LNM versus PT; red, positive-to-negative switch in LNM compared to PT, yellow no change in expression; white, unable to analyze sample.

No	*TWIST1* mRNA	*SNAIL* mRNA	*SLUG* mRNA	TWIST1	SNAIL	SLUG	ER	PgR	HER2	VIM	E-CAD
264											
235											
308											
332											
297											
313											
263											
284											
276											
285											
283											
255											
280											
295											
341											
224											
206											
289											
334											
267											
247											
278											
322											
299											
311											
307											
273											
288											
310											
331											
336											
291											
306											
200											
326											
242											
243											
252											
294											
328											
296											
253											
223											
203											
**Conversion (-) -> (+)**	**9/29**	**3/29**	**6/29**	**10/39**	**17/38**	**7/39**	**6/40**	**9/41**	**1/34**	**1/42**	**0**
**%**	**31**	**10**	**21**	**26**	**45**	**18**	**15**	**22**	**3**	**2**	**0**
**Conversion (+) -> (-)**	**6/29**	**5/29**	**7/29**	**8/39**	**3/38**	**4/39**	**1/40**	**3/41**	**0**	**2/42**	**0**
**%**	**21**	**18**	**24**	**20**	**8**	**10**	**3**	**7**	**0**	**5**	**0**
**Total conversion**	**15/29**	**8/29**	**13/29**	**18/39**	**20/38**	**11/39**	**7/40**	**12/41**	**1/34**	**3/42**	**0**
**%**	**52**	**28**	**45**	**46**	**53**	**28**	**18**	**29**	**3**	**7**	**0**
