# Supplementary material for: Expression of epithelial to mesenchymal transition-related markers in lymph node metastases as a surrogate for primary tumor metastatic potential in breast cancer
Source: J Transl Med. 2012 Nov 19;10:226. doi: 10.1186/1479-5876-10-226 (PMC3524044; doi:10.1186/1479-5876-10-226)
Supplement: Additional file 3 — Figure S3. Immunohistochemical staining of EMT-related markers in lymph nodes metastases. Exemplary results of negative and positive staining of E-cadherin, vimentin, TWIST1, SLUG and SNAIL. [file 1479-5876-10-226-S3.pdf]

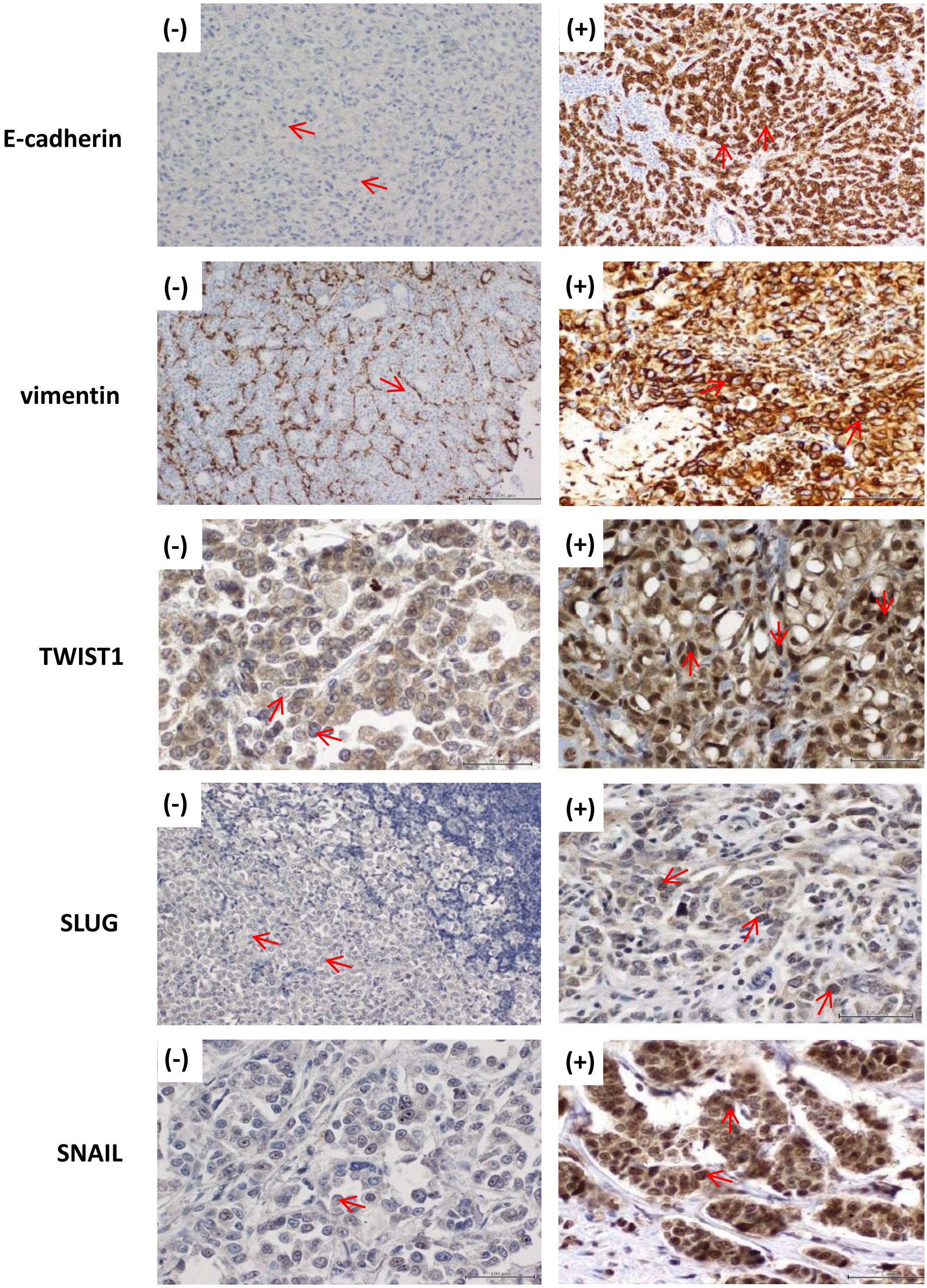

Figure 3S. Immunohistochemial staining of EMT-related markers in lymph nodes metastases. Exemplary results of negative and positive staining of E-cadherin (membrane staining), vimentin (cytoplasmic staining), TWIST1 (nuclear staining), SLUG (nuclear staining) and SNAIL (nuclear staining). Arrows indicate tumor cells either negative or positive for a particular protein on respective photos. Immunostaining of SNAIL produced cytoplasmic background staining in some cells, but only cells with nuclear protein accumulation indicative of transcriptional activity were considered positive.
