# Supplementary material for: Expression of epithelial to mesenchymal transition-related markers in lymph node metastases as a surrogate for primary tumor metastatic potential in breast cancer
Source: J Transl Med. 2012 Nov 19;10:226. doi: 10.1186/1479-5876-10-226 (PMC3524044; doi:10.1186/1479-5876-10-226)
Supplement: Additional file 4 — Figure S4. Immunohistochemical staining of receptors in lymph nodes metastases. Exemplary results of negative and positive staining of estrogene receptor (ER), progesterone receptor (PgR) and human epidermal growth factor receptor 2 (HER2). [file 1479-5876-10-226-S4.pdf]

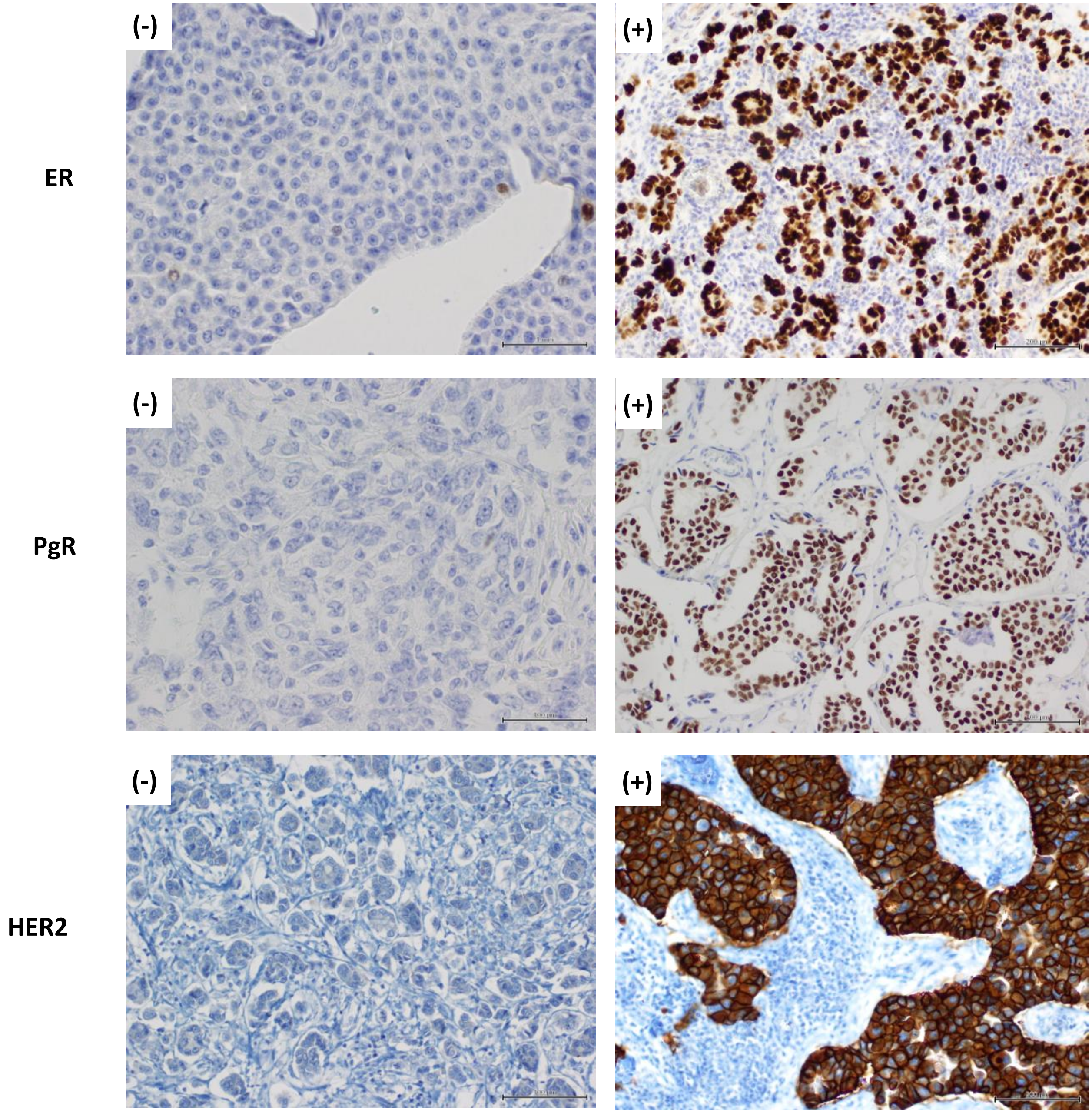

Figure 4S. Immunohistochemial staining of receptors in lymph nodes metastases. Exemplary results of negative and positive staining of estrogene receptor (ER), progesterone receptor (PgR) and human epidermal growth factor receptor 2 (HER2).
