# Supplementary material for: Expression of epithelial to mesenchymal transition-related markers in lymph node metastases as a surrogate for primary tumor metastatic potential in breast cancer
Source: J Transl Med. 2012 Nov 19;10:226. doi: 10.1186/1479-5876-10-226 (PMC3524044; doi:10.1186/1479-5876-10-226)
Supplement: Additional file 5 — Table S2. Correlation between the expression of EMT-transcription factors and the number of involved lymph nodes. Results for both mRNA and protein levels of TWIST1, SNAIL and SLUG are presented. [file 1479-5876-10-226-S5.pdf]

Table 2S. Correlation between the expression of EMT-transcription factors and the number of involved lymph nodes. Results for both mRNA and protein levels of TWIST1, SNAIL and SLUG are presented. χ^2 or Fisher's exact test were used where appropriate. P≤0.05 was considered statistically significant.

PT – primary tumor, LNM – lymph nodes metastases, N – number of samples, NS – not significant.

	Analyzed marker	Expression status	Number of involved lymph nodes		N	P
			**≤3**	**>3**		
mRNA	***TWIST 1* PT**	negative	11	2	13	NS
		positive	9	6	15	
	***SNAIL* PT**	negative	10	4	14	NS
		positive	12	4	16	
	***SLUG* PT**	negative	9	3	12	NS
		positive	13	5	18	
	***TWIST1* LNM**	negative	7	6	13	0.07
		positive	5	17	22	
	***SNAIL* LNM**	negative	7	10	17	NS
		positive	5	14	19	
	***SLUG* LNM**	negative	7	10	17	NS
		positive	5	14	19	
protein	**TWIST1 PT**	negative	8	15	23	NS
		positive	7	9	16	
	**SNAIL PT**	negative	8	15	23	NS
		positive	7	8	15	
	**SLUG PT**	negative	12	17	29	NS
		positive	4	7	11	
	**TWIST1 LNM**	negative	11	10	21	**0.02**
		positive	3	15	18	
	**SNAIL LNM**	negative	6	4	10	0.07
		positive	8	21	29	
	**SLUG LNM**	negative	12	19	31	NS
		positive	2	5	7	
